# More Data, Please: Machine Learning to Advance the Multidisciplinary Science of Human Sociochemistry

**DOI:** 10.3389/fpsyg.2020.581701

**Published:** 2020-10-22

**Authors:** Jasper H. B. de Groot, Ilja Croijmans, Monique A. M. Smeets

**Affiliations:** ^1^Faculty of Social and Behavioural Sciences, Utrecht University, Utrecht, Netherlands; ^2^Behavioural Science Institute, Radboud University, Nijmegen, Netherlands

**Keywords:** sense of smell, machine learning, chemosignals, non-verbal communication, social and personality psychology

## Abstract

Communication constitutes the core of human life. A large portion of our everyday social interactions is non-verbal. Of the sensory modalities we use for non-verbal communication, olfaction (i.e., the sense of smell) is often considered the most enigmatic medium. Outside of our awareness, smells provide information about our identity, emotions, gender, mate compatibility, illness, and potentially more. Yet, body odors are astonishingly complex, with their composition being influenced by various factors. Is there a chemical basis of olfactory communication? Can we identify molecules predictive of psychological states and traits? We propose that answering these questions requires integrating two disciplines: psychology and chemistry. This new field, coined *sociochemistry*, faces new challenges emerging from the sheer amount of factors causing variability in *chemical composition* of body odorants on the one hand (e.g., diet, hygiene, skin bacteria, hormones, genes), and variability in *psychological* states and traits on the other (e.g., genes, culture, hormones, internal state, context). In past research, the reality of these high-dimensional data has been reduced in an attempt to isolate unidimensional factors in small, homogenous samples under tightly controlled settings. Here, we propose big data approaches to establish novel links between chemical and psychological data on a large scale from heterogeneous samples in ecologically valid settings. This approach would increase our grip on the way chemical signals non-verbally and subconsciously affect our social lives across contexts.

## Introduction

Humans are surprisingly good smellers. The pervasive myth that humans are only “tiny smellers” has been debunked by 21st century research showing a wide array of smell skills (Stevenson, 2010; de Groot et al., 2017; McGann, 2017). To name a few: humans can follow a scent-trail (like sniffer dogs; Porter et al., 2007), detect certain odorants at extremely low levels (few droplets in an Olympic size swimming pool; Yeshurun and Sobel, 2010), and identify diseases like Parkinson’s before actual diagnosis (Trivedi et al., 2019). In our everyday lives, smells have a “communicative” function, informing us about the quality of food and warning us for environmental hazards (e.g., gas leaks) (Stevenson, 2010). An even less well-known function of smell is *social* communication (de Groot et al., 2017; Parma et al., 2017; Pause, 2017; Roberts et al., 2020); the topic of this article. Studies have shown that our smells provide others with cues about our identity and gender (Penn et al., 2007), age (Mitro et al., 2012), health (Olsson et al., 2014), and emotions (de Groot et al., 2015; Pause et al., 2020). This form of communication occurs without our voluntary control and generally outside of our awareness, which imbues chemical communication with mystery. Demystifying the spreading of social information through smell was listed in *Science* as one of the 125 most compelling multidisciplinary puzzles facing scientists this century (Kennedy and Norman, 2005). Our goal here is to outline *how* researchers could go about answering this query, whether there is a universal “language” of social smells. Society at large will be helped by optimally leveraging fundamental insights emerging from this view to worldwide industrial and clinical applications that could improve a person’s quality of life.

Social smells are markedly complex: body odor contains thousands of molecules (de Lacy Costello et al., 2014), and massive variability is caused by factors including genotype, hormonal status, mood, skin bacteria, diet, smoking, hygiene habits, clothing, and use of fragranced products (e.g., Natsch and Emter, 2020; Roberts et al., 2020). Past studies have generally sidestepped this challenge by performing small-scale psychological experiments under carefully controlled, sterile conditions (for a meta-analysis: de Groot and Smeets, 2017; for a critical view: Wyatt, 2020). These studies formed the first stepping stones by strongly suggesting that social information can be communicated via smell under tightly controlled settings; yet, (i) the molecules transmitting the message have generally remained elusive, as well as (ii) the ecological settings in which chemical communication occurs. Not dealing with these obstacles could deadlock future research efforts to “anchor” molecules to their social source. To accelerate future research, we propose (i) multidisciplinary ways of working by integrating psychology and chemistry toward a science of human *sociochemistry* (Box 1), and (ii) moving outside of the sterile lab to test subjects with diverse backgrounds.

**Box 1. Definition of sociochemistry.**

With sociochemistry we refer to the *multidisciplinary science* examining non-verbal social communication via human body odor, particularly focusing on the chemistry between people.

The sociochemistry we advance is a multidisciplinary, ecological approach that in view of its inherent complexity requires an ecosystem of academic institutions around the world to flourish, by working together to create speed and scale (cf. Forscher et al., 2020). We propose the building of open access databases holding information that spans across chemistry (e.g., chemical composition of sweat odor) and psychology (e.g., capturing the states and traits of those participating in the chemosignaling as well as their unique contextual information. Machine learning techniques can be applied to generate models that may accurately predict molecules’ sway on our social lives across diverse contexts and samples, with technological, societal, and clinical applications following suit.

In what follows, we will first outline the initial research questions, methods, and advances of past research, before we will identify current obstacles to a broader and deeper understanding of human chemical communication, and we will end with a perspective on how to overcome these hurdles in future research.

## Past Research: Simplifying a Complex Problem

Communication is crucial to humans. Most of our communication is non-verbal. Of all the sensory channels engaged in non-verbal communication, smells arguably pose the biggest deciphering challenge.

The past research in this field initially focused on determining *what* social information can be communicated via smell. To test this, researchers have systematically attempted to eradicate “noise” on the chemical communication channel by controlling extraneous factors (e.g., diet, hygiene, fragranced product use) and testing homogeneous samples in carefully controlled lab experiments. In these studies, sweat was collected from *senders* (who had kept to a scent-free regimen for multiple days to isolate the experimentally-induced chemical “message”) and presented to *receivers* in a separate experiment. Chemical communication was inferred from recipients’ behavioral, affective, physiological, neuroendocrine and/or neural responses matching the sender’s state. This way, numerous double-blind experiments showed that human smells can convey information from fleeting emotions and sickness, to more enduring traits like identity, gender, reproductive status, and age (for reviews, see de Groot et al., 2017; Parma et al., 2017; Pause, 2017).

Although past research on human chemical communication has provided initial insights into the type of information human odors can bring across, we identify a number of obstacles for a better (quicker, broader, and deeper) understanding of non-verbal communication via smell.

### Problem I: Small Scale, Slow Speed

The current science of non-verbal communication via smell is rooted in a longstanding tradition of strictly controlled laboratory experiments focusing on the empirical testing of hypotheses addressing cause-effect relations, using reliable and validated methods and carefully calibrated instruments (for empirical demonstrations, see e.g., Chen and Haviland-Jones, 2000; Regenbogen et al., 2017; Endevelt-Shapira et al., 2018; Quintana et al., 2019; de Groot et al., 2020b; Gomes et al., 2020; Pause et al., 2020 (for recent narrative overviews, see e.g., Loos et al., 2019; Ferdenzi et al., 2020; Havlíček et al., 2020 (for meta-analyses, see e.g., Gildersleeve et al., 2014; de Groot and Smeets, 2017). This approach, with a preference for intrinsic over extrinsic validity, has been the method of choice to build our (psychological) science for decades. Despite advantages of scientific rigor and quality, there are problems in speed and scale. With little coordination across labs around the world, different researchers may be working on similar research questions (e.g., “can humans smell fear?”; Mujica-Parodi et al., 2009; Pause et al., 2009; Prehn-Kristensen et al., 2009; Zhou and Chen, 2009), each moving through the laborious cycle of recruiting, screening, and testing senders and receivers with barely sufficient statistical power (as outlined by Wyatt, 2020). The essence to our argument is that there is a stark contrast between the *complexity* of the problem at the root of sociochemistry, which is the mystery of the correspondence between the chemical “code” and the message it carries on the one hand, and the relatively slow tactic of churning experiments one at a time.

### Problem II: Generality of Findings

Second, we need to characterize the generality of findings or extrinsic validity of the traditional experiments (Simons et al., 2017). Both uniformity in subject characteristics and test settings form obstacles to a broader understanding of the potentially species-wide and real-world impact of non-verbal communication via smell. Open queries include: Is the language of smell universal? How much of this communication is modified by context, a powerful moderating factor in olfactory science (e.g., Dalton, 1999; De Araujo et al., 2005; de Groot et al., 2020a)? Can this language be “heard” beyond the thick walls of labs, in noisy field settings? Answering these questions will help chart the impact of social smells on the daily lives of many.

Because past research has been typified by (i) context-deprived lab experiments, presenting (ii) uncontaminated sweat samples, using (iii) a relatively small number of subjects with (iv) relatively uniform characteristics, we currently have no knowledge of how broadly shared human olfactory communication is. To illustrate, the male-to-female chemical communication dyad initially served to increase experimental sensitivity, with males generally having the larger and more active sweat glands, and females being the slightly better smellers (but see this meta-analysis: Sorokowski et al., 2019; and this review: Majid et al., 2017, for gender differences that are at most small and affecting only higher order smell processing). Although initially useful, this gender uniformity adds a constraint on generality, and the same goes for the almost exclusive reliance on participants that are Western, Educated, Industrialized, Rich, Democratic (WEIRD; Henrich et al., 2010) (cf. de Groot et al., 2018; Roberts et al., 2020). Generalizing research findings from WEIRD samples to other populations is a major problem in science in general, and a particularly pressing issue when one examines the breadth and scale of the non-verbal language of smells (Box 2).

**Box 2. Sociochemical language.**

A sociochemical language would imply configurations of chemical symbols that convey meaning, which meaning is acquired via learning. This notion of language would acknowledge the possibility that (i) identical chemical configurations do not mean the same to everyone, (ii) the meaning of an identical chemical configuration may vary even to a single individual depending on context, (iii) that there is (substantial) variation or “noise” around one chemical configuration, from which one single uniform meaning can still be distilled. Therefore, the language would not have to be universal.

### Problem III: Unidisciplinary Research

Third, to be able to forge a link between smell molecules and behavior we need to move beyond a single-discipline research tradition. Although several psychological studies have revealed systematic patterns in the behaviors of senders and recipients (in relatively sterile, uniform settings), the *chemical message* driving this coupling has generally remained enciphered (but see Penn et al., 2007; Smeets et al., 2020). Lessons can be learned from the animal literature, where the combination of rigorous behavioral experiments (bioassay) and chemical analysis (isolating, identifying, and synthesizing the bioactive substance to recreate the bioassay-behavior) forms the golden standard to detect a common chemical “language” for a species: *pheromones* (Wyatt, 2015, 2020). But the definition of pheromones, rooted in entomological research as single molecules eliciting innate responses in a conspecific (Karlson and Lüscher, 1959), appears outdated and unsuitable for mammals like humans, as our smell perception strongly depends on learning and context, and our body emits a multitude of molecules (de Groot et al., 2017). The minimum pragmatic evidence, however, is to determine (in a collaborative, multi-lab effort) whether human chemical language is *consistent in form* (requiring a multidisciplinary approach) and *broadly shared* across the human species (requiring diverse samples and settings).

## Prospective Advances

In the wake of recent developments in psychological research and theory, chemical analytical technology, and data science (discussed below), substantial progress can be made now to unravel the symbol system of social smell. Specifically, we outline an integration of traditional psychology methods and chemistry toward a new science of human sociochemistry, studying human chemosignaling across various ecologically valid settings and samples, across all human diversity. To deal with the complexity and large, multidimensional databases that emerge from this interdisciplinary, ecologically valid endeavor, we propose applying data science approaches like machine learning. We anticipate that large scale multidisciplinary collaborations are required to get us closer to identifying the alphabet of the language of social smells and assess its real-world impact.

### Multidisciplinary Approach: Deciphering the Alphabet of Social Smells

Any attempt to get closer to the answer of whether social smells convey a common language requires a multidisciplinary combination of psychological experiments and chemical analysis.

Most research on human chemical communication focused on psychological effects. The few studies that did apply chemical analysis have shown that certain characteristics and transient emotions could be identified in a sender’s body odor. One pioneering study by Penn et al. (2007) showed that a person’s identity and gender could be expressed in a person’s body odor, with 14 molecules predicting gender with 75% accuracy. Based on remarkable anecdotal evidence that a human “super smeller” could detect Parkinson’s Disease (PD) by smell, Trivedi et al. (2019) found that four compounds (eicosane, hippuric acid, octadecanal, and perillic aldehyde) were characteristic markers of PD; when smelling these compounds, the super smeller subjectively reported a strong PD smell. Other studies found chemical markers suggestive of fear (and happiness). Potential chemical markers for fear were identified by in armpit odor (Smeets et al., 2020), stress levels were also expressed in a person’s breath (Preti et al., 2019; acetone, isoprene, dimethyl sulfide), and in a creative field study, (Williams et al., 2016) showed that scary and funny film events reliably changed the emission of molecules from cinema audiences. Taken together, these multidisciplinary studies show the potential for social information to be encoded in a person’s smell in predictable ways, thus jumpstarting a sociochemistry approach to identify a common smell language.

Whereas on the one end of the scientific spectrum, we have this classic tradition of sequentially conducting laboratory experiments designed to address a specific causal hypothesis derived from theory, carefully controlling for measurement error and extraneous influence. On the other end there is the big data approach relying on machine learning analytical techniques performed on big databases holding what seems to be unrelated information from large populations to magically reveal unexpected correlations unencumbered by theory (Mayer-Schönberger and Cukier, 2013). Neither, on its own, will be an optimal path for unraveling human sociochemistry and the underlying language on which it is built. What we propose, instead, is a hybrid approach, a combination in which machine learning techniques are used to help us find handles on and insights into the composition of the chemical signal combinations that are the building blocks of the signal, and the related individual and external variables to further sculp this unique form of social communication. These insights will contribute to the formulation of hypotheses about cause and effect that can then be isolated and tested in controlled lab environments (cf. Wyatt, 2015, 2020).

### Ecological Validity: A Broadly Shared, Widely Used Social Smell Language?

In the quest for discovering a potential universal language of smell that is also societally relevant, we argue that the highest success rate can be achieved by first examining smells whose detection generally aids survival (Schaal and Porter, 1991).

In the earliest stages of life, when vision and hearing are still underdeveloped, the smell of mothers’ milk is a powerful cue that attracts a newborn to the food source (Schaal et al., 2020). Even formula-fed newborns oriented more toward the smell of an unfamiliar lactating woman than to the familiar formula smell (Porter et al., 1991); and this was not a novelty effect, as the same smell was also preferred over the breast odor of nulliparous women (Makin and Porter, 1989). There may well be universal chemical cues in the breast odor of lactating women that attract most if not all newborns under diverse ecologically valid settings, but this still requires empirical investigation from non-WEIRD samples (Schaal et al., 2020).

Humans would also benefit from picking up smells indicating danger, like fear sweat threatening physical harm, and disease sweat threatening contamination. The capacity to register these invisible, far-reaching, and long-lasting chemical warning cues would have increased our ancestors’ survival chances. Indeed, the smell of fear has been shown to instigate adaptive processes: a fearful facial expression (raised eyebrows, opened nose) and increased sensory intake (eyes and nose) to better detect threat (de Groot et al., 2012); yet, typically this phenomenon has not been examined beyond WEIRD samples, with one East Asian exception (de Groot et al., 2018). Quintana and colleagues (2019) further assessed the breadth of chemical communication in a controlled yet ecologically valid Virtual Reality environment. They found that smelling fear/stress sweat induced anxiety in recipients and reduced their interpersonal trust toward a virtual character. Even outside of the lab, the smell of fear (masked in clove odor, making it undetectable) could negatively impact dental student performance (Singh et al., 2018). Indeed, odor masking (e.g., with perfume, deodorant) could not prevent recipients from making consistent and reliable smell-based social judgments at typical social distances (Gaby and Zayas, 2017). Taken together, these findings allude to fear/stress smell affecting behavior across contexts in diverse samples, but more data is needed.

The complex and resource-intensive methodology of sweat sampling and exposure has arguably held back large (field) experiments, but upscaling and including natural settings seems inevitable in an attempt to discover the commonalities in human smells and their practical application, with big data approaches providing structure within the anticipated wealth of transdisciplinary data.

### Machine Learning: Solving the Big Data Challenge Ahead of Us

In vision and hearing, the wavelength of light and frequency of sound are highly predictive of color and tone; yet, predicting the smell of a molecule from its chemical structure is much harder. In the past decade, researchers have started using machine learning techniques to demonstrate links between molecular structure and odor perception (for an overview: Lötsch et al., 2018). Machine learning, a popular application of artificial intelligence, is a set of methods that can be used to automatically detect patterns in data and use these patterns to predict or classify future data (e.g., Murphy, 2012; Dhar, 2013). Although machine learning models have shown the feasibility of predicting odor perception from relatively simple, non-social smells (Khan et al., 2007; Zarzo, 2011; Snitz et al., 2013; Keller et al., 2017; Gutiérrez et al., 2018; Sanchez-Lengeling et al., 2019) a number of extra challenges emerge when machine learning is applied to uncover the language of social smells. The difference between past “non-social” models and what we propose here is that (i) past models predicted odor perception from physico-chemical properties of *single* chemical compounds, whereas body odors are mixtures of compounds, and the communicative signal also likely having a multi-component architecture (Loos et al., 2014), the composition of which requires employing chemical analytical techniques to elucidate; (ii) past model endpoints have traditionally been *sensory endpoints* (e.g., intensity, pleasantness, and qualitative descriptors like garlicky or fruity) as opposed to social-behavioral endpoints (e.g., perceivers’ affect, physiology, behavior); (iii), past models have not considered various sample characteristics (excepting gene variants coding for odorants receptors) or ecologically relevant contexts that are expected to impact smell perception as well.

**FIGURE 1:**
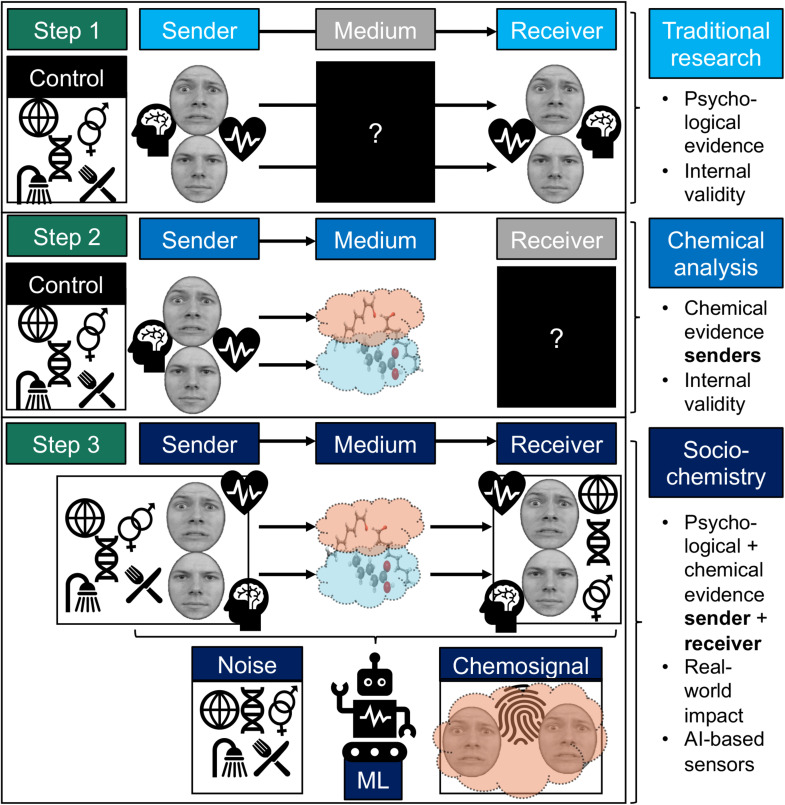
Possible pathway to understanding sociochemistry using machine learning (ML). The different steps denote past/present (Step 1 and 2) and proposed (Step 3) approaches to elucidate human social communication via smell. The steps are increasingly data-intense and complex and go from uni- to multidisciplinary research. Although there is no strict order, Step 1 and 2 can form initial building blocks for sociochemistry (Step 3), by testing psychological correspondence between senders and receivers in traditional ways (Step 1; chemical medium “remains” black box); and by decoding psychological/clinical information from the sender’s smell (Step 2; the receiver’s response and therefore the *social* chemosignal remains “black box”). Controlling for various factors (e.g., genotype, culture, gender, hygiene, diet) is recommended here to initially isolate the signal and/or its psychological effect. However, true chemical communication (e.g., of emotions like fear) involves (i) studying behavioral/physiological/brain response patterns in senders *and* receivers, while (ii) identifying the molecules that *link* two humans (i.e., the social (chemo)signal in human-human interaction), (iii) under ecologically valid conditions (i.e., including “noise” factors like dietary and hygiene habits) (Step 3), to eventually develop artificial intelligence-based sensors that could be applied in the real world for senders (e.g., diagnosis) and receivers (e.g., facilitating well-being by blocking the signals from entering the nose). This is an example of fear chemosignaling (vs. neutral), using faces obtained from the Radboud Faces Database (Langner et al., 2010).

To identify human chemosignals within the vast amount of data that can encompass body odors (a big data challenge), we recommend moving away from using a single, traditional statistical model (e.g., logistic regression), and instead propose a sequence of different analyses, including machine learning (ML). It would seem premature to rigorously define each step in the analysis sequence, but we will sketch a possible analysis “pipeline” (Figure 1):

**Step 1** would entail collecting sweat from senders induced to be in a particular state (e.g., fear, happiness, disgust, sickness) or having a characteristic of interest (e.g., gender, personality, genotype). A subset of these sweat samples would then be used as *stimuli* in another experiment involving human receivers, whose behavioral responses will form a benchmark for verifying effective chemical communication (requiring a sender and receiver).

In **Step 2**, the remaining sweat samples will be used for chemical analysis. After extracting the molecules using headspace, solvent, or direct extraction techniques, chemical analysis could entail two-dimensional gas chromatography-mass spectrometry (GCxGC-ToF-MS) allowing for comprehensive profiling of the volatile molecules in the sweat samples and their discriminative power between two (or more) states/traits of interest. Because there is little to no background knowledge on chemical classes associated with presumed signals in sweat odor, initial research by Smeets et al. (2020) used *untargeted* screening approaches to distinguish between fear, happiness, and a neutral state, and found a matrix of over a 1,000 chemical volatile peaks. This number could be reduced as a next step to 94 by selecting only those peak intensities that differed significantly with at least one other emotion category. Preprocessing the GC × GC-ToF-MS profiles into total-intensity-count values (TICs) is another way to yield a smaller, more manageable subset of peaks of interest (cf. Lebanov et al., 2020). What could further ease the future identification of unique chemical profiles predicting human states/traits are *templates* (reference peak profiles) that follow from overlaying all chromatograms in a set (cf. Stilo et al., 2019), or using previous datasets as templates (cf. Reichenbach et al., 2019). This requires acquiring large chemical datasets, which necessitates high-throughput approaches like automated extraction and (ultra)fast GCxGC-ToF-MS, followed by automated quantification of specific target compounds belonging to specific states and traits.

In **Step 3**, ML techniques could help identify the core chemical features of human states/traits in multiple ways. Unsupervised learning (e.g., k-means clustering) could yield potentially interesting clusters of chemicals that are involved in chemical communication not considered before. Supervised learning could be applied next by training an algorithm on a large subset of samples, and testing the trained model on the remaining set. While there are vast varieties in learning algorithms, they can broadly be divided into linear or non-linear based on the shape of the decision surface used to classify data. Linear methods, like support vector machines (SVM) with linear kernels, may be preferred because they perform at least on par with non-linear methods (e.g., Misaki et al., 2010, in the context of separating emotions with fMRI data) while remaining straightforward to interpret. The interpretability of the models from the pipeline we propose might be tested by comparing the predictive power of those models with the outcomes on receiver experiments. To illustrate, Reichenbach et al. (2019) combined GC × GC chemical profiling with SVM to predict different characteristics of wines (e.g., grape variety, origin). Although the wines had considerable overlap in their chemical composition (up to 25% overlap in grape variety), the analysis yielded a number of highly distinctive molecules that the models used to differentiate the wines with around 90% accuracy (Reichenbach et al., 2019). At the same time, the resulting models were still relatively intuitively interpretable (cf. Mori and Uchihira, 2019).

We believe that applying a chemosignal-identification pipeline as described above would also yield relatively straightforward models, with an interpretable set of chemical predictors that are highly predictive of the emotions under investigation. Feature selection in our machine learning pipeline could be based on, depending on the ML technique used, mean absolute error (MAE) of the predictor (in case of regression-based techniques) or area under the curve (AUC) measures (in case of classification-based techniques) (Molnar, 2018). Selection of the best performing predictors could be tested by application level evaluation (cf. Molnar, 2018), using follow-up lab or crowd-sourced experiments where the most likely molecule candidates are tested in appropriate molecular concentrations.

## Database-Building: Back to the Future

The proposed analysis pipeline requires rather large, well-populated databases compared to current standards. At present, there is a lack of such (publicly available) databases. Ideally, data in these databases contain a vast amount of parameters from hundreds of participants (senders *and* receivers). These parameters include personal factors (e.g., gender, age, country of residence, genotype), lifestyle factors (e.g., deodorant use, hygiene habits), measures of context (e.g., sterile lab vs. field), health, personality, and emotion (e.g., subjectively reported emotions and psychophysiological measures), and thousands of additional parameters per sample resulting from chemical analysis. Hence, the complexity and vastness of the resulting database underscores the need to step away from experimenter-driven analyses techniques such as traditional regression models, and turn to automatic feature selecting analyzation algorithms instead. Using these ML techniques has another advantage – the possibility to directly apply the best performing models in artificial intelligence applications.

However, one big hurdle to take with this multivariate, machine learning approach is the need for large, ecologically valid datasets. Talking about big data, a now famous 1989 National Geographic Smell Survey managed to test and analyze data from 1.42 million respondents to examine the relation between olfaction and aging (Wysocki and Gilbert, 1989). In a related effort to build socially relevant smell databases, Snitz et al. (2019) cleverly combined online crowdsourcing with the physical distribution of “scratch-and-sniff” odorants (via regular mail), and collected data (now publicly available) from about 1,000 individuals in 100 days. In these studies, chemical communication had not been the focus. If body odors were the topic, the database should hold matrices of (i) the chemical constituents of body odors (alongside multivariate information about, e.g., the emotional state during which the body odor was produced), as well as (ii) relevant person- and situation-specific variables of senders donating the samples (e.g., diet, hygiene product use, culture, genetic variation), and (iii) person- and situation-specific variables of recipients.

Acquiring such a complex and elaborate database can impossibly be a single-lab endeavor, and in our view, coordination within a larger ecosystem of labs will be crucial. Fortunately, technical advances in communication allow large consortia of researchers to globally collaborate from the comfort of their homes, like the Global Consortium for Chemosensory Research (GCCR), which focuses on the relation between COVID-19 and chemosensory dysfunction. Within weeks, hundreds of researchers around the globe collaborated to design an online study resulting in a large (open access) database on COVID-19 and smell/taste dysfunction and a published manuscript (Parma et al., 2020). Moreover, in work that focused on smell communication (literally, being able to talk about smells), Majid et al. (2018) have examined for 20 languages whether there exists a universal hierarchy to vision being more accessible to consciousness and linguistic description than smell. These, and other examples (e.g., Iravani et al., 2020), have illustrated that global consortia can be instrumental in acquiring the necessary datasets to solve complex and urgent questions in a timely manner.

## Technology Supporting Societal and Clinical Impact

We envision the application of machine learning to understand human non-verbal communication to yield a series of impactful consequences ranging from psychology to medicine. If machine learning techniques can pick up on statistical regularities between, for instance, emotional states and health conditions on the one hand and patterns of molecules on the other, chemical sensors can be developed to read this “smell language” in real time. Promisingly, Imam and Cleland (2020) placed an array of 72 chemosensors (based on the architecture of the mammalian olfactory bulb) in a wind tunnel, which rapidly learned and identified odor representations, despite various sources of noise. Given that body odor (itself susceptible to noise) contains information about emotions (Smeets et al., 2020) and one’s health, ranging from markers for Parkinson’s disease (Trivedi et al., 2019), general inflammatory reactions (Olsson et al., 2014), to possibly the presence of COVID-19 in sweat (Grandjean et al., 2020), it would be intriguing to explore whether such algorithms could be used to learn and identify the even more complex language inherent to human odors.

The near future could see a rapid growth in the diagnostic implementation of sweat odor analysis that could happen outside of a lab or clinic in a person’s home, with the emergence of novel smartphone-based biosensors (Brasier and Eckstein, 2019). Through these smartphone-based on-skin biosensors, sweat compounds could become broadly available in databases as digital biomarkers. Such an in-home approach is expected to have a major influence on clinical and outpatient care, and could even prevent infectious diseases from spreading by suggesting self-quarantining. The impact of these biosensors may extend to therapeutic settings, where the smell-based detection of patients’ emotions (or lack thereof) could provide an insightful role in (online) therapeutic sessions. In sum, physicians and clinicians could foresee their instrumentation being expanded in the future by sensors and machine learning to more quickly, accurately, and safely get a grip on a disease or clinical problems and their prognosis (Chen et al., 2019).

## Conclusion

Although scientific evidence has shown that the sense of smell serves a number of crucial functions in the daily life of humans, including social communication (e.g., Stevenson, 2010; de Groot et al., 2017; McGann, 2017), the idea that humans are micro smellers has remained hardwired among scientists and laypeople. However, through smell, humans can (unwillingly) convey information about a person. These initial advances were generally obtained under the most sterile conditions, by single research groups from the perspective of a single discipline. Although initially fruitful, we caution that continuing this experimental tradition will stall scientific progress toward a broader, deeper, and quicker understanding of non-verbal communication via smell. In the quest for discovering the real-world impact of social smells in diverse samples across diverse settings, we focused on the importance of ecological testing conditions, multidisciplinary research, and open collaborations to populate high dimensional databases, with machine learning approaches “making sense” of the complicated statistical regularities between smell molecules and physical or psychological conditions (the science of sociochemistry). By informing us about food, danger, health, and hygiene, olfaction serves a crucial role in human life, and so much so, that losing our sense of smell dramatically reduces the quality of our life. Our invitation for a better fundamental and practical understanding of the language of human smells opens up a multitude of (technological) possibilities, including tailor-made or world-wide clinical and societal applications proportionate to the scale at which human odors non-verbally communicate information from a sender to a recipient, whether human or machine.

## Data Availability Statement

The original contributions presented in the study are included in the article/supplementary material, further inquiries can be directed to the corresponding author.

## Author Contributions

All authors were involved in the conceptualization of this research. JG drafted the outline of this manuscript, wrote the manuscript, and edited the manuscript. IC and MS co-wrote the manuscript and critically revised the outline of the manuscript and the manuscript itself.

## Conflict of Interest

The authors declare that the research was conducted in the absence of any commercial or financial relationships that could be construed as a potential conflict of interest.
